# Early Screen Exposure and Preadolescent Outcomes: A Longitudinal Follow-Up on Dysregulation, Academic Achievements, and Capacity to Be Alone

**DOI:** 10.3390/children12111544

**Published:** 2025-11-14

**Authors:** Luca Cerniglia, Silvia Cimino

**Affiliations:** 1 Faculty of Psychology, International Telematic University Uninettuno, 00186 Rome, Italy; 2 Department of Dynamic, Clinical and Health Psychology, Sapienza, University of Rome, 00185 Rome, Italy; silvia.cimino@uniroma1.it

**Keywords:** screen exposure, dysregulation, academic results, capacity to be alone

## Abstract

**Highlights:**

**What are the main findings?**

- Early screen exposure at age 4 contributed to persistent dysregulation through age 12, which in turn mediated lower academic achievement and reduced capacity to be alone.
- Maternal scaffolding buffered the early effects of screen time on dysregulation, though this protective role was strongest at age 6 and diminished thereafter.

**What is the implication of the main finding?**

- Early digital media use can set in motion developmental cascades that undermine both academic adjustment and introspective capacities, underscoring the long-term risks of excessive screen exposure.
- Preventive strategies should integrate screen-time guidelines with parenting interventions that strengthen scaffolding behaviors to promote children’s self-regulation and resilience.

**Abstract:**

Background: Previous longitudinal evidence suggested that screen exposure at age 4 was associated with dysregulation symptoms and lower academic achievement up to age 8. Yet, it remains unclear whether these effects persist in preadolescence and extend to higher-order developmental outcomes such as the capacity to be alone, a marker of self-regulation and autonomy within the developmental psychopathology framework. Aim: This follow-up study re-contacted the original cohort at age 12 (T3) to examine whether early screen time predicted dysregulation, academic achievement, and capacity to be alone, testing the mediating role of dysregulation at ages 6 (T1) and 8 (T2), and the moderating role of maternal scaffolding at age 4. Methods: A community sample of N = 323 children and their mothers, previously assessed at T0–T2, was re-evaluated at T3 (mean age = 12.2 years, SD = 0.7). At T0, screen exposure and maternal scaffolding were measured using the StimQ (PIDA subscale). Dysregulation at T1–T3 was assessed with the Teacher Report Form (TRF). Academic achievement in mathematics and literacy was rated by teachers using the Teacher Academic Ratings. At T3, children also completed the Capacity to Be Alone Scale for Children (CBASC). Structural Equation Modeling (SEM) tested longitudinal direct, indirect, and moderated pathways, adjusting for sex, maternal education, and socioeconomic status. Results: Screen time at age 4 was associated with elevated dysregulation at T1 and T2, which in turn mediated poorer mathematics and literacy outcomes and reduced capacity to be alone at age 12 (all *p* < 0.01). Maternal scaffolding buffered early dysregulation but did not prevent long-term academic or self-regulatory impairments. Conclusions: Findings indicate that early excessive screen use contributes to a cumulative cascade of dysregulation, undermining both academic achievement and the developmental capacity to be alone by preadolescence. Preventive strategies should integrate screen-time guidelines with parental scaffolding interventions.

## 1. Introduction

Digital technology has become ubiquitous in early childhood, raising questions about its long-term impact on development. Young children today are exposed to smartphones, tablets, and other screens from infancy, often far exceeding the modest limits suggested by pediatric experts [1,2]. While interactive media can provide educational content, excessive screen time in the preschool years is widely believed to displace crucial developmental activities such as hands-on play, face-to-face social interaction, and self-directed exploration [3,4]. Indeed, a growing body of research links high early screen exposure with adverse outcomes in multiple domains. For example, prolonged screen use in early childhood has been associated with sleep disruptions, delayed language development, and poorer executive functioning, as well as increases in externalizing and internalizing behavior problems [5,6]. These correlations align with the hypothesis that heavy reliance on screens may undermine children’s developing capacity for self-regulation and sustained attention, although the magnitude and causal direction of these effects remain topics of ongoing investigation [7].

Within this context, Cerniglia and colleagues [8] conducted a longitudinal study highlighting the potential long-term risks of excessive screen time in early childhood. Following a cohort of 422 children from age 4 to age 8, they reported that children who had greater exposure to digital devices at 4 years old showed significantly higher levels of emotion dysregulation and lower academic achievement by 8 years old. In particular, early screen use was a direct predictor of later difficulties with self-regulation and poorer performance in foundational school subjects such as literacy and mathematics. These findings echo other recent reports that link high screen use with socio-emotional and cognitive challenges in childhood. For instance, a 2025 survey of school-age children in India found that total screen time was negatively correlated with academic grades and positively correlated with anxiety symptoms and behavior problems [9,10]. Similarly, experimental and short-term longitudinal evidence suggests that when parents habitually use digital devices to pacify upset toddlers, children show heightened emotional reactivity and poorer self-control over time [11]. Taken together, these studies support a growing concern that abundant screen exposure in the early years may hinder the development of children’s internal mechanisms for emotion regulation and learning, potentially setting them on a maladaptive developmental trajectory.

Another important dimension concerns the role of parental support in early development, particularly maternal scaffolding. Maternal scaffolding refers to the caregiver’s ability to guide and structure the child’s activity through verbal cues, modeling, and emotional support. This interactive style helps children develop self-regulation and autonomy by providing external regulation that gradually becomes internalized [12,13]. In the context of early screen exposure, scaffolding may act as a protective factor, helping children manage stimulation and maintain attention during media use. Conversely, low levels of scaffolding may increase vulnerability to dysregulation, as children receive fewer co-regulated experiences that promote emotional and behavioral control. For these reasons, maternal scaffolding represents a key variable in understanding individual differences in children’s responses to early digital media exposure [13].

A developmental psychopathology framework offers a useful theoretical lens for understanding these findings and for guiding further inquiry. This perspective emphasizes examining how early-life experiences and individual differences interact over time to shape either adaptive or maladaptive outcomes [14]. From this viewpoint, early excessive screen use could be conceptualized as a risk factor that may initiate cascading effects on development. For example, difficulties with emotion regulation emerging in middle childhood (around age 6–8) could carry forward into later periods, especially if the child encounters new challenges without having acquired effective coping skills. On the other hand, developmental psychopathology also attends to questions of discontinuity and multifinality – not all children with early screen exposure will follow the same path [15]. Some may adapt or compensate as they grow older, depending on protective factors in their environment (such as supportive parenting, high-quality schooling, or individual resilience). Thus, an important next step is to extend longitudinal research on early screen time to later developmental stages, to determine whether the early-emerging differences observed by age 8 persist, worsen, or possibly attenuate by preadolescence. By following the original cohort into preadolescence, we can examine the continuity of early effects and also identify any new domains of adjustment that might be implicated.

Preadolescence (around 11–12 years of age) represents a pivotal transition period that is ideal for such a follow-up. At this age, children are on the cusp of adolescence and undergo significant changes biologically, cognitively, and socially. They enter puberty, move toward more independent roles at school and with peers, and develop more complex self-identities. These changes may both exacerbate earlier vulnerabilities and present new challenges that were not salient in middle childhood. For instance, a child who had difficulty with emotion regulation at age 8 might struggle even more at age 12 when faced with the intensified social pressures of early adolescence. Moreover, digital media use often increases substantially by preadolescence—many 12-year-olds have their own smartphones or frequent access to social media and online entertainment [16]. This means that any habits or dependencies formed in early childhood could be amplified in a more permissive media environment. In light of these considerations, re-contacting the cohort at age 12 provides a critical opportunity to observe how early screen-related patterns interact with the new developmental tasks of preadolescence. By age 12, one can assess not only academic progress and behavioral adjustment in school, but also emerging psychosocial competencies that are important for adolescent well-being.

One such competency, and the focal new outcome in the present extension of the study, is the child’s capacity to be alone. The capacity to be alone refers to an individual’s ability to tolerate and even appreciate solitude without distress, maintaining a sense of contentment and self-regulation in the absence of external stimuli or social interaction. First articulated by Winnicott [17] as a hallmark of emotional maturity, this capacity reflects the developmental attainment of internalized security and coping skills. A child with a strong capacity to be alone can spend time by themselves—entertaining their own thoughts or engaging in solitary play—without excessive boredom, anxiety, or dysphoria. In contrast, children with a low capacity to be alone may feel intense discomfort or agitation when not occupied by external engagement, often seeking immediate stimulation or reassurance from screens or others. The current digital era has raised concerns that constant access to devices might impede the development of this skill; children today are rarely “alone with their thoughts” if smartphones, tablets, or televisions are always within reach [18]. Some scholars worry that this phenomenon of solitude deprivation could deprive young people of the formative experiences of boredom and self-reflection that previous generations used to learn how to self-soothe and creatively occupy themselves [19]. From a developmental psychopathology perspective, the capacity to be alone can be seen as a protective factor that helps individuals navigate stress and negative emotions. Recent studies with adolescents support this idea, showing that those with greater capacity for solitude tend to have better psychological outcomes. For example, in one longitudinal survey, higher capacity to be alone was associated with lower subsequent levels of depression and anxiety among youth, even after accounting for other positive personal characteristics [20]. Although the standardized effects were modest in size (βs ranging from 0.14 to 0.20), they are meaningful in developmental and educational terms. For instance, a β = 0.14 from early screen exposure to teacher-rated dysregulation corresponds approximately to a 0.25–0.30 SD increase in the dysregulation score for each additional hour of daily screen use at age 4. Given that teacher-rated dysregulation predicts later emotional and academic difficulties, even small shifts in this magnitude can have meaningful implications at the population level. Another study during the COVID-19 pandemic found that adolescents with a strong capacity to be alone were less vulnerable to emotional problems when cut off from usual social supports; in fact, this capacity buffered the impact of both high social media use and poor parent–child relationship quality on youths’ psychopathological symptoms [8]. These findings suggest that the ability to tolerate solitude confers resilience in the face of stressors and may mitigate the harmful effects of excessive screen engagement or social isolation.

Despite its apparent importance, the capacity to be alone has seldom been examined in relation to early childhood experiences. Little is known about what developmental precursors might foster or impede the growth of this capacity. It is plausible that early excessive screen exposure could be one such precursor: children who become accustomed to constant digital entertainment from toddlerhood onward may have fewer opportunities to practice being alone without stimulation. If a preschooler learns to rely on a tablet or smartphone whenever they feel bored or upset, they might be less inclined to develop independent coping strategies (such as imaginative play, daydreaming, or other self-soothing behaviors) that enhance comfort with solitude. Over time, this could manifest as an impaired capacity to be alone by preadolescence, wherein the child feels restless or dysregulated in the absence of a screen or external engagement. The extension of the Cerniglia et al. [8] longitudinal study to age 12 enables us to empirically investigate this hypothesis. In the present study (Wave 4 of the longitudinal project), we assess the original cohort’s capacity to be alone at age 12 and examine its associations with early screen exposure and previously measured outcomes. Using the developmental psychopathology framework, we will evaluate whether the trajectory from early screen use to later dysregulation and academic outcomes extends into the domain of preadolescent solitude capacity. We will also explore the stability or change in the earlier observed relationships: for instance, do children who showed dysregulated behavior or lower academic achievement by age 8 continue to exhibit these difficulties at age 12, or has the gap widened/narrowed? By re-engaging this cohort at the cusp of adolescence, the study seeks to deepen our understanding of how early digital experiences relate to later developmental competencies and adjustment. Such knowledge is not only scientifically valuable in mapping the course of digital-age development, but also practically important. If early screen habits have enduring or cascading effects into preadolescence, this would underscore the need for early preventive guidance for families. Conversely, if some early effects dissipate or are moderated by later experiences, identifying those moderating factors (e.g., parenting practices, peer influences, or individual traits like the capacity to be alone) can inform more nuanced interventions. In summary, extending this longitudinal study to include a fourth wave at age 12 allows us to integrate and build upon previous findings by examining a new, developmentally salient outcome through the lens of developmental psychopathology. The introduction of capacity to be alone as a dependent variable at preadolescence broadens the scope of inquiry from academic and behavioral outcomes to the child’s inner emotional resilience. This extension is important because it captures an aspect of psychosocial functioning especially relevant in our technology-saturated era. The present study, therefore, not only replicates and extends the earlier findings on dysregulation and academic achievement but also breaks new ground by linking early screen exposure to the preadolescent ability to manage solitude. In doing so, it aims to contribute to a more comprehensive understanding of how early digital environments shape child development over time, ultimately informing strategies to promote healthy outcomes in the context of ubiquitous technology.

The present study had four specific objectives:

1. To examine continuity of dysregulation: We aimed to test whether early screen exposure at age 4 (T0) was associated higher levels of dysregulation at ages 6 (T1), 8 (T2), and 12 (T3), extending prior findings to preadolescence.
2. To assess academic trajectories: We investigated whether early screen exposure indirectly was associated lower academic achievement in literacy and mathematics at ages 6, 8, and 12 through its association with dysregulation across development.
3. To introduce a novel developmental outcome: We evaluated whether early screen exposure was associated children’s capacity to be alone at age 12, either directly or indirectly via dysregulation and academic achievement.
4. To test the moderating role of maternal scaffolding: Finally, we examined whether maternal scaffolding at age 4 buffered the association between early screen exposure and subsequent dysregulation, replicating and extending moderation effects observed in earlier waves.

Based on the above literature we made the following hypotheses:

**H1.**  *Early screen exposure at age 4 (T0) will positively contribute to dysregulation across development, such that children with higher screen exposure will show higher dysregulation at ages 6 (T1), 8 (T2), and 12 (T3).*

**H2.**  *Early screen exposure will be negatively associated with children’s academic achievement in literacy and mathematics, and this effect will be mediated by dysregulation at T1–T2. Specifically, higher early screen exposure will be associated with greater dysregulation, which in turn will predict lower academic performance through preadolescence.*

**H3.**  *Early screen exposure will negatively contribute to children’s capacity to be alone at age 12 (T3). This effect will occur both directly and indirectly, via the mediating role of dysregulation and academic achievement.*

**H4.**  *Maternal scaffolding at age 4 will moderate the association between early screen exposure and dysregulation at T1. The positive association between early screen exposure and dysregulation will be weaker among children whose mothers reported higher levels of scaffolding.*

## 2. Materials and Methods

### 2.1. Sample and Procedure

This study represents the fourth wave (T3) of an ongoing longitudinal project that originally began when children were 4 years old (T0). Earlier findings from T0–T2, focusing on outcomes up to age 8, have been reported elsewhere [8]. The present paper reports new data collected when the same cohort was re-contacted at age 12, while also drawing on previously collected measures for longitudinal modeling. Importantly, the results presented here are based on the newly gathered T3 assessments; prior data are included only as predictors within the longitudinal framework. At T3, 323 families (Mage child = 12.2 years, SD = 0.7; 52% female) participated, representing 76.5% of the original cohort. Families who did not take part at T3 were lost due to relocation, refusal, or inability to be contacted. Attrition analyses indicated no significant differences between retained and non-retained families on key baseline variables (sex, maternal education, screen exposure, and dysregulation) (Table 1).

Data collection at T3 included: (a) teacher reports of children’s dysregulation and academic achievement, paralleling earlier assessments at T1 and T2; and (b) a new child self-report instrument measuring the capacity to be alone, introduced for the first time at this wave. Baseline measures of screen exposure and maternal scaffolding (T0), along with teacher-rated dysregulation and academic achievement (T1–T2), were incorporated into structural models to test longitudinal pathways.

All procedures were conducted in school settings by trained research assistants blind to study hypotheses. Parents provided renewed written informed consent for T3, and children gave verbal assent. Ethical approval for the study was granted by the Institutional Review Board of [University anonymized for review].

Data were collected in four waves approximately two years apart. Baseline assessments (T0) took place in 2014, when children were about 4 years old. Follow-ups were conducted in 2016 (T1; age 6), 2018 (T2; age 8), and 2022 (T3; age 12). The interval between T2 and T3 was longer than in previous waves due to organizational delays and the COVID-19 pandemic, which also coincided with part of the T3 data collection. This contextual factor is acknowledged in the Discussion.

### 2.2. Measures

At baseline (T0; child age 4), mothers reported their child’s average daily time spent with four types of digital devices: television/video, smartphone, tablet, and computer. These items were administered alongside the StimQ–PIDA battery but were developed specifically for this study to quantify children’s digital media exposure. They are therefore not part of the original PIDA scoring, which focuses on parental scaffolding behaviors. For each device, mothers indicated the typical number of hours per day their child used it (open numeric response, recorded to the nearest 0.5 h). Reported hours across devices were summed to compute a total daily screen exposure index (hours/day). Values above the 99th percentile were winsorized to limit extreme outliers while maintaining rank order. The resulting distribution was slightly right-skewed, consistent with previous research on children’s screen use. The untransformed total score had a mean of 3.10 h/day (SD = 1.20; N = 323). Analyses were also run using a log1p-transformed variable (log[hours + 1]), yielding equivalent results; thus, the raw variable was retained for interpretability.

Maternal scaffolding was assessed at T0 using the StimQ–PIDA subscale [21], a structured parent-report measure designed to capture the extent to which caregivers support children’s learning and problem-solving during everyday interactions. The adapted version used in this study included items on activities such as labeling objects, counting, or guiding digital play to promote the child’s cognitive engagement. Higher scores reflected more frequent and elaborative scaffolding behaviors.

In order to assess child dysregulation, teachers completed the Teacher Report Form (TRF; [22] at T1 (age 6), T2 (age 8), and T3 (age 12). Following standard procedures, the Dysregulation Profile was derived by combining the Anxious/Depressed, Aggressive Behavior, and Attention Problems subscales. The TRF has established reliability and validity in Italian samples, with higher scores indicating greater difficulties in behavioral and emotional regulation.

For academic achievement, teachers at T1–T3 filled out the Teacher Academic Ratings scale [23]. This instrument asks teachers to evaluate children’s performance in literacy and mathematics on a 5-point Likert scale (1 = far below grade level, 5 = well above grade level). Domain-specific mean scores were computed for literacy and mathematics. Because children progressed through different grades, teachers varied across waves. However, school transitions followed the standard educational pathway, and no systematic differences in participation or ratings were observed across classrooms or schools.

Finally, to evaluate capacity to be alone at T3, children completed the Capacity to Be Alone Scale for Children (CBASC; [24]). This self-report questionnaire includes items rated on a 4-point Likert scale (1 = not at all true, 4 = very true), assessing dimensions such as comfort with solitude, self-soothing, and the positive use of solitary time. The CBASC has been shown to possess a unidimensional factor structure and satisfactory psychometric validity in school-age samples, including Italian children. Higher scores indicate a greater ability to tolerate and benefit from being alone. In the present study, internal consistency was satisfactory (Cronbach’s α = 0.76).

### 2.3. Analyses Plan

All analyses were conducted using structural equation modeling (SEM) in Mplus Version 8 [25]. To accommodate the mild positive skew of the screen-time variable, all models were estimated using maximum likelihood with robust standard errors (MLR). Sensitivity analyses using the log-transformed screen exposure variable (log[hours + 1]) yielded equivalent results, supporting the robustness of the findings. We specified a longitudinal path model to examine whether screen exposure at age 4 (T0) predicted later outcomes in dysregulation, academic achievement, and capacity to be alone at age 12 (T3). Dysregulation scores at T1 (age 6) and T2 (age 8) were included as mediators, allowing us to test indirect pathways linking early screen use to subsequent outcomes. In addition, maternal scaffolding at T0 was modeled as a moderator of the association between early screen exposure and dysregulation at T1, following the approach adopted in the prior wave of this project. Moderation was tested by creating a latent product term (Screen Exposure × Maternal Scaffolding) within the Latent Moderated Structural Equations (LMS) framework in Mplus.

To account for shared variance across constructs, all outcome variables were allowed to correlate at each time point. Child sex, maternal education, and socioeconomic status were entered as covariates on all endogenous variables. Missing data were handled using full-information maximum likelihood (FIML), which yields unbiased estimates under the assumption of missing at random. The missingness pattern was inspected using Little’s MCAR test and descriptive analyses, indicating that data were consistent with the missing-at-random (MAR) assumption. Model fit was evaluated using multiple indices: the comparative fit index (CFI) and Tucker–Lewis index (TLI), with values ≥ 0.95 considered excellent, and the root mean square error of approximation (RMSEA) and standardized root mean square residual (SRMR), with values ≤ 0.06 and ≤0.08, respectively, indicating good fit [26].

**Figure 1:**
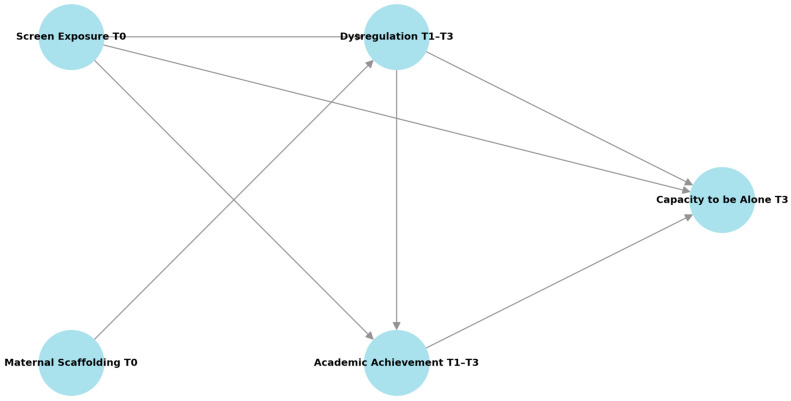
Model tested with SEM specified screen exposure at age 4 (T0) as an exogenous predictor of children’s dysregulation (T1–T3), academic achievement (T1–T3), and capacity to be alone (T3). Maternal scaffolding at T0 was included as a moderator of the pathway from screen exposure to dysregulation at T1, using the latent moderated structural equations (LMS) approach. Dysregulation trajectories were modeled as sequential mediators, transmitting the effect of early screen exposure to later academic achievement and capacity to be alone. Child sex, maternal education, and socioeconomic status were included as covariates on all endogenous variables. Model fit was evaluated using CFI, TLI, RMSEA, and SRMR, following [26] criteria.

Indirect effects were tested using bias-corrected bootstrapped confidence intervals based on 5000 resamples. Significant mediation was inferred when the 95% confidence interval did not include zero. Interaction terms for moderation analyses were estimated using the latent moderated structural equations (LMS) method, and simple slope analyses were performed to interpret significant effects. Attrition analyses (retained vs. lost families) revealed no significant baseline differences in sociodemographic or key study variables (all ps > 0.10, |d| < 0.20). Missing data were addressed using FIML under a missing-at-random assumption (Little’s MCAR test n.s.). Prior power simulations for comparable SEM models indicate that our sample (N = 323) provides sufficient power (>0.80) to detect moderate direct and interaction effects (β ≈ 0.20−0.25). The latent interaction between screen exposure and maternal scaffolding was tested using the Latent Moderated Structural Equations (LMS) approach in Mplus Version 8 (TYPE = RANDOM; ESTIMATOR = MLR). Because standard global fit indices (e.g., CFI, TLI, RMSEA) are not available for LMS models, we compared the model loglikelihood and information criteria (AIC, BIC, SABIC) with the additive model (no interaction) to evaluate improvement in fit. Figure 1.

## 3. Results

Preliminary analyses examined descriptive statistics and intercorrelations among all study variables (see Table 2). As expected, early screen exposure was positively correlated with dysregulation across time points and negatively correlated with academic achievement and capacity to be alone. Maternal scaffolding was inversely related to dysregulation and positively associated with later outcomes.

The hypothesized SEM showed good overall fit to the data, χ^2^(245) = 318.72, *p* < 0.001, CFI = 0.963, TLI = 0.951, RMSEA = 0.041 (90% CI = 0.033−0.049), SRMR = 0.039, indicating that the proposed model adequately captured the observed relations.

As predicted (H1), children’s screen exposure at age 4 was a robust predictor of subsequent dysregulation. Higher early screen use was associated with greater dysregulation at age 6 (β = 0.28, SE = 0.07, z = 4.01, *p* < 0.001) and age 8 (β = 0.21, SE = 0.08, z = 2.63, *p* = 0.009). This association persisted, though at a weaker level, at age 12 (β = 0.14, SE = 0.06, z = 2.21, *p* = 0.027), supporting the idea of continuity in dysregulatory difficulties across development.

With respect to academic performance (H2), the direct links between early screen exposure and teachers’ ratings of literacy and mathematics were nonsignificant at all time points (all ps > 0.25). However, significant indirect effects emerged: screen exposure predicted lower achievement at age 8 via dysregulation at age 6 (indirect β = −0.11, 95% CI [−0.18, −0.05]) and lower achievement at age 12 through dysregulation across middle childhood (indirect β = −0.14, 95% CI [−0.22, −0.07]). These findings indicate that the effect of early screen time on school outcomes operates largely through dysregulation processes rather than directly.

Turning to the novel outcome introduced in this study (H3), early screen exposure also predicted children’s self-reported capacity to be alone at age 12. Higher screen exposure was associated with reduced capacity to be alone (β = −0.17, SE = 0.07, z = −2.51, *p* = 0.012). Again, dysregulation emerged as a mediator: children exposed to more screen time at age 4 showed greater dysregulation at ages 6–8, which in turn was linked to lower scores on the Capacity to Be Alone Scale at age 12 (indirect β = −0.09, 95% CI [−0.15, −0.03]). In contrast, academic achievement did not significantly mediate this association (indirect β = −0.02, 95% CI [−0.06, 0.01]).

**Figure 2:**
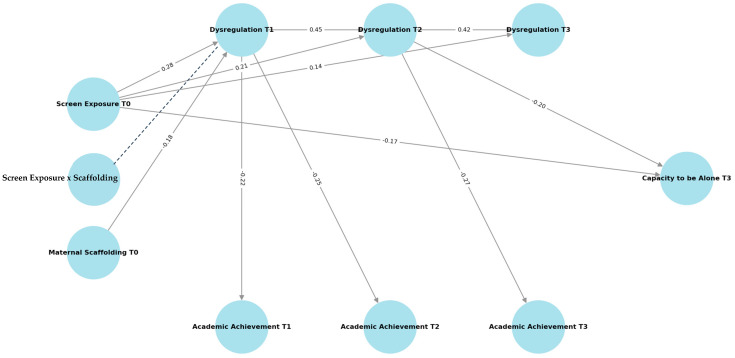
Structural equation model linking early screen exposure to dysregulation, academic achievement, and capacity to be alone. Note. Standardized coefficients (β) are shown on significant paths. Early screen exposure at age 4 (T0) predicted higher dysregulation at ages 6–12, which in turn was associated with lower academic achievement at ages 6–12 and reduced capacity to be alone at age 12 (T3). Maternal scaffolding at T0 negatively predicted dysregulation at age 6, buffering the effect of early screen exposure. Covariates (child sex, maternal education, socioeconomic status) and nonsignificant paths are omitted for clarity. Solid lines indicate significant direct paths; the dashed line represents the latent interaction term (Screen Exposure × Maternal Scaffolding) tested via the LMS moderation model in Mplus. All paths shown are standardized (β) and significant at *p* < 0.05.

Finally, analyses tested the buffering role of maternal scaffolding (H4). The interaction was modeled as a latent product term (Screen Exposure × Maternal Scaffolding) within the LMS framework in Mplus. The latent interaction significantly predicted dysregulation at age 6 (β = −0.13, SE = 0.06, z = −2.19, *p* = 0.028), indicating that maternal scaffolding moderated the association between early screen exposure and later dysregulation. Simple slope analyses showed that the positive effect of screen exposure on dysregulation was strong when scaffolding was low (simple slope = 0.34, *p* < 0.001), moderate when scaffolding was average (simple slope = 0.19, *p* = 0.018), and nonsignificant when scaffolding was high (simple slope = 0.05, *p* = 0.47). However, no significant moderation effects emerged for later outcomes at ages 8 or 12. Table 3 presents the results of the path analyses, including direct, indirect, and interaction effects. As shown, early screen exposure significantly predicted increases in dysregulation across time points, with subsequent indirect effects on academic achievement and capacity to be alone. In addition, maternal scaffolding moderated the association between early screen exposure and dysregulation at T1. Figure 2.

## 4. Discussion

The present study extends previous work on the developmental consequences of early digital media use [8] by adding a new assessment point in preadolescence and incorporating the construct of capacity to be alone. While the earlier study demonstrated that screen exposure at age 4 contributed to dysregulation and early academic difficulties up to age 8, the current findings provide evidence that such trajectories persist up to age 12 and broaden their scope to include relational and introspective outcomes. In doing so, this study not only replicates but also expands prior results, thereby strengthening the argument that early screen habits can have enduring implications for children’s adjustment.

The first and perhaps most robust finding of this study is that higher exposure to digital screens at age 4 contributed to a persistent trajectory of emotional and behavioral dysregulation up to early adolescence. This result aligns with previous longitudinal evidence showing that excessive screen time in preschool years is associated with later socioemotional difficulties, including poor attentional control, heightened irritability, and increased externalizing behaviors [27]. By extending these trajectories until age 12, our findings suggest that the negative impact of early screen exposure may not be transient but can consolidate into more stable regulatory difficulties. These results are consistent with ecological models of development emphasizing the displacement hypothesis, whereby digital media use replaces experiences crucial for self-regulation such as imaginative play, physical activity, and emotionally attuned interactions with caregivers [28,29].

At the same time, it is important to acknowledge that some studies have reported more nuanced associations, highlighting that the effects of digital media depend on content, context, and the presence of co-viewing caregivers [30]. For example, interactive educational programs may not entail the same risks as unsupervised or entertainment-only screen use, and in some cases may even contribute to socio-cognitive growth. The current study did not differentiate by type of screen content, which may partly explain differences with reports of null or even beneficial associations in other samples [31]. Nevertheless, the overall convergence with longitudinal evidence suggests that early, heavy exposure to screens constitutes a risk factor for later dysregulation, particularly when not embedded in scaffolding relationships.

Beyond its impact on socioemotional adjustment, our findings indicate that early screen exposure exerts an indirect influence on children’s academic performance through the pathway of dysregulation. Specifically, difficulties in emotional and behavioral regulation served as a mediator between screen time at age 4 and later literacy and mathematics outcomes, highlighting that it is not the mere exposure to digital devices that undermines school achievement, but the enduring regulatory impairments that such exposure fosters. This interpretation is in line with developmental frameworks that conceptualize self-regulation as a foundational competence for academic learning, supporting children’s capacity to sustain attention, manage frustration, and engage effectively with complex cognitive tasks [32].

These findings extend the results of our previous work [8], which already suggested that screen exposure was associated with early academic challenges via regulatory difficulties. By following the cohort into preadolescence, the present study confirms that such indirect effects persist over time, thereby reinforcing the hypothesis of a cascading developmental mechanism. Comparable results have been reported in broader longitudinal samples, where executive function and self-regulation in early childhood consistently contributed to later achievement in core school domains [33,34]. Our data suggest that excessive early screen use may jeopardize these crucial regulatory capacities, thus indirectly affecting academic success.

Nonetheless, it is worth noting that some studies have documented direct associations between screen exposure and poorer school performance, without mediation by regulation [35]. Such discrepancies may reflect differences in measurement (e.g., teacher-reported vs. standardized achievement tests), or in the type of media consumed. For instance, the predominance of passive entertainment content may more directly displace learning opportunities, whereas interactive or educational media may have more nuanced effects. By focusing on the mediating role of dysregulation, our study contributes to clarifying this debate, suggesting that regulation difficulties are a primary mechanism through which early media use shapes academic trajectories.

A novel contribution of the present study concerns the association between early screen exposure and children’s capacity to be alone at age 12. This construct, originally conceptualized in psychoanalytic and developmental traditions [17], refers to the ability to tolerate solitude, engage in reflective self-experience, and regulate affect without constant external stimulation. Our results indicate that greater exposure to screens in preschool years was associated with a reduced ability to be alone in preadolescence, suggesting that early reliance on digital devices may hinder the development of internal resources for self-soothing and introspection.

This finding resonates with emerging concerns in the literature that pervasive digital stimulation may limit children’s opportunities to cultivate imaginative play, boredom tolerance, and autonomous coping strategies [36]. From this perspective, screen time may not only displace external interactions but also interfere with the child’s relationship with the self, reducing the likelihood of developing comfort in solitude. Although the concept of capacity to be alone has received little empirical attention in the context of media research, related evidence supports the idea that heavy media use is linked to difficulties in emotion regulation and to heightened dependence on external sources of stimulation [37].

At the same time, the present findings should be interpreted cautiously. The measure of capacity to be alone captures a multifaceted psychological construct, and the mechanisms linking early screen use to this outcome remain speculative. One possibility is that continuous external stimulation prevents children from experiencing and mastering moments of solitude during sensitive developmental windows, thereby curtailing opportunities to internalize regulatory experiences. Alternatively, the association may be partly bidirectional: children who are temperamentally less comfortable with solitude may be more drawn to screens, thereby reinforcing this vulnerability. Future research should explore these dynamics longitudinally and with more fine-grained measures of digital use and introspective capacity.

Finally, the moderating effect of maternal scaffolding provides an important protective perspective within this developmental picture. Consistent with the statistical model, the latent interaction between screen exposure and maternal scaffolding (tested via LMS) significantly predicted dysregulation at age 6, confirming that scaffolding buffered the association between early screen use and regulatory difficulties. Our analyses showed that high levels of maternal scaffolding at age 4 attenuated the association between early screen exposure and subsequent dysregulation, suggesting that parental support can buffer the potential risks associated with digital media use. This result is consistent with a body of research demonstrating that sensitive and structured parental engagement fosters the development of regulatory skills, even in the context of environmental or individual vulnerabilities [38,39].

From an ecological standpoint, this finding underscores that screen exposure is not inherently detrimental but exerts its most negative effects in the absence of supportive caregiving. Scaffolding behaviors—such as guiding attention, encouraging problem-solving, and providing emotional support—likely enhance children’s ability to cope with stimulation and frustration, counterbalancing the dysregulating influence of early digital overuse. Similar protective effects have been reported in studies showing that parental mediation of media use reduces associations between screen time and negative outcomes, such as sleep disruption or behavioral difficulties [40,41].

At the same time, the protective role of scaffolding may be developmentally constrained. In our data, moderation effects were most evident at age 6, suggesting that parental scaffolding is particularly influential during early school years, when children are still consolidating basic self-regulatory competences. This pattern suggests a sensitive period during the early school years, when parental scaffolding may exert greater proximal influence on children’s regulatory development, whereas later adjustment is increasingly shaped by peer and school contexts. As children grow older and spend more time outside of the parental sphere, scaffolding may have diminishing direct impact, leaving regulatory trajectories more strongly determined by earlier experiences. This temporal specificity points to the importance of interventions aimed at supporting parents in the preschool years, when scaffolding may be most effective in buffering risks associated with early digital exposure.

Taken together, the moderating effect of scaffolding highlights that the risks associated with digital media are neither uniform nor inevitable. Rather, they are embedded in a broader relational ecology, where sensitive parental practices can either mitigate or amplify developmental vulnerabilities.

The strengths of this study include its prospective longitudinal design, the multi-informant assessment of regulatory difficulties and outcomes, and the integration of both socioemotional and introspective constructs such as capacity to be alone. These features allow for a nuanced examination of how early screen habits shape development across multiple domains.

However, several limitations must be acknowledged. First, the study relied on maternal reports for several key measures at later time points, raising the possibility of perceptual bias. Second, maternal scaffolding was assessed through a brief parent-report subscale, which captures the frequency but not the qualitative dimensions of scaffolding behaviors. Future research could include observational or multi-informant measures to provide a more comprehensive understanding of this construct. Third, the capacity to be alone was assessed only once, at age 12, preventing conclusions about its developmental trajectory or earlier antecedents. Fourth, we did not assess the content or context of screen exposure—such as educational versus entertainment use, or solitary versus co-viewing conditions—which limits the specificity of interpretations, as these factors may differentially influence outcomes. Fifth, the sample was culturally circumscribed to families in Rome, potentially limiting generalizability to more diverse populations and cultural settings.

Moreover, while the longitudinal design allows for stronger inferences than cross-sectional studies, causality cannot be firmly established, and unmeasured third variables (e.g., child temperament, paternal involvement) may partly account for the observed associations. It should also be noted that data collection at T3 (2022) partly overlapped with the COVID-19 pandemic, which affected schooling and daily routines in Italy. Although preliminary checks did not reveal systematic differences in participation or key variables, this contextual factor may have influenced children’s screen use and self-regulatory experiences. Future studies should consider how such large-scale disruptions may interact with developmental processes across time. Although more complex cross-lagged or RI-CLPM models could further separate within- and between-person effects, our design and construct coverage did not allow for such specifications. Furthermore, because parental mental health and child temperament were not available at baseline, we could not account for these potentially confounding influences. Future studies should include such variables to better isolate environmental from dispositional effects.

Another consideration concerns possible alternative explanations for the observed associations between early screen exposure and later dysregulation. Although our analyses focused on the moderating role of maternal scaffolding, other child and family characteristics may contribute to these pathways. For instance, children with more difficult temperamental traits—such as high negative affectivity or low effortful control—may both elicit greater screen use from parents and show higher baseline dysregulation, partially accounting for the longitudinal associations observed. Similarly, family routines and daily structure (e.g., consistency of meal and sleep schedules, shared activities) may influence both exposure to digital media and the development of self-regulatory skills. Future research should incorporate such dispositional and contextual variables to disentangle environmental effects from individual differences in susceptibility to dysregulation.

Despite these limitations, the present findings carry important implications. They underscore the need for preventive interventions that address both child and parent behaviors: limiting excessive screen use in preschool years, supporting children in developing self-regulation skills, and empowering parents to scaffold children’s regulatory capacities. Moreover, the inclusion of capacity to be alone opens promising avenues for research and practice, suggesting that fostering children’s comfort with solitude may be an overlooked target for interventions in the digital age. These conclusions align with recent meta-analytic evidence highlighting small but consistent associations between screen use and children’s socio-emotional and cognitive outcomes, and the moderating role of family context and parental involvement [42].

## Figures and Tables

**Table 1:** Socio-demographic characteristics of the sample.

Variable	M	SD	%	N
Child age (years)	12.2	0.7	–	
Sex (female)	–	–	52	
Maternal education (years)	14.5	2.8	–	
Maternal ethnicity (white) Household income (>39,900 euros/year) (%)			98 82	
Screen exposure at age 4 (h/day)	3.1	1.2	–	
Maternal scaffolding	2.95	0.75		323

**Table 2:** Means, standard deviations, and intercorrelations among study variables.

Variable	1	2	3	4	5	6	7	8	9
1. Screen exposure T0	–								
2. Dysregulation T1	0.38	–							
3. Dysregulation T2	0.12	0.13	–						
4. Dysregulation T3	0.13	0.48	0.15	–					
5. Academic achievement T1	−0.29	−0.49	−0.39	−0.30	−				
6. Academic achievement T2	−0.26	−0.05	−0.31	−0.08	−0.22	–			
7. Academic achievement T3	−0.10	−0.22	−0.23	−0.29	0.41	−0.18	–		
8. Capacity to be alone T3	−0.08	−0.21	−0.19	−0.15	0.01	0.07	0.20	–	
9. Maternal Scaffolding T0	−0.09	−0.21	−0.19	−0.15	0.01	0.07	0.20	0.34	

Note. Values represent Pearson correlation coefficients (r). Two-tailed significance: *p* < 0.05, *p* < 0.01, *p* < 0.001. All variables measured at child ages 4 (T0), 6 (T1), 8 (T2), and 12 (T3).

**Table 3:** Direct, indirect, and interaction effects of early screen exposure on dysregulation, academic achievement, and capacity to be alone across time (standardized coefficients).

Predictor → Outcome	β	SE	z	*p*	95% CI
Direct effects					
Screen exposure T0 → Dysregulation T1	0.28	0.07	40.01	<0.001	[0.15, 0.41]
Screen exposure T0 → Dysregulation T2	0.21	0.08	20.63	0.009	[0.05, 0.36]
Screen exposure T0 → Dysregulation T3	0.14	0.06	20.21	0.027	[0.02, 0.26]
Screen exposure T0 → Academic achievement T1	−0.07	0.06	−10.12	0.26	[−0.19, 0.05]
Screen exposure T0 → Academic achievement T2	−0.05	0.07	−00.71	0.48	[−0.18, 0.09]
Screen exposure T0 → Academic achievement T3	−0.06	0.08	−00.77	0.44	[−0.21, 0.09]
Screen exposure T0 → Capacity to be alone T3	−0.17	0.07	−20.51	0.012	[−0.30, −0.04]
Indirect effects (bootstrapped)					
Screen exposure T0 → Achiev. T2 via Dysreg. T1	−0.11	–	–	–	[−0.18, −0.05]
Screen exposure T0 → Achiev. T3 via Dysreg. T1–T2	−0.14	–	–	–	[−0.22, −0.07]
Screen exposure T0 → Capacity T3 via Dysreg. T1–T2	−0.09	–	–	–	[−0.15, −0.03]
Screen exposure T0 → Capacity T3 via Achiev. T1–T2	−0.02	–	–	–	[−0.06, 0.01]
Moderation (interaction effects)					
Screen exposure × Scaffolding T0 → Dysreg. T1	−0.13	0.06	−20.19	0.028	[−0.25, −0.01]

Note. β = standardized coefficient. Indirect effects tested with 5000 bias-corrected bootstraps; 95% CI reported.

## Data Availability

Data are available upon reasonable request to the corresponding author, in accordance with institutional and ethical regulations.
